# Review of functional MRI in HIV: effects of aging and medication

**DOI:** 10.1007/s13365-016-0483-y

**Published:** 2016-10-07

**Authors:** C. S. Hakkers, J. E. Arends, R. E. Barth, S. Du Plessis, A. I. M. Hoepelman, M. Vink

**Affiliations:** 1 Department of Internal Medicine and Infectious Diseases, University Medical Center Utrecht, Heidelberglaan 100, 3508 GA Utrecht, The Netherlands; 2 Department of Psychiatry, University of Stellenbosch, Cape Town, South Africa; 3 Department of Psychiatry, University Medical Center Utrecht, Heidelberglaan 100, 3508 GA Utrecht, The Netherlands

**Keywords:** HAND, fMRI, BOLD, Systematic review

## Abstract

HIV-associated neurocognitive disorder (HAND) is a frequently occurring comorbidity of HIV infection. Evidence suggests this condition starts subclinical before a progression to a symptomatic stage. Blood oxygenated level dependent (BOLD) fMRI has shown to be a sensitive tool to detect abnormal brain function in an early stage and might therefore be useful to evaluate the effect of HIV infection on brain function. An extensive literature search was performed in June 2015. Eligibility criteria for included studies were as follows: (1) conducting with HIV-positive patients, (2) using BOLD fMRI, and (3) including a HIV-negative control group. A total of 19 studies were included in the review including 931 participants. Differences in activation between HIV-positive and -negative participants were found when testing multiple domains, i.e., attention, (working) memory, and especially executive functioning. Overall, HIV-positive patients showed hyperactivation in task-related brain regions despite equal performances as controls. Task performance was degraded only for the most complex tasks. A few studies investigated the effect of aging on fMRI, and most of them found no interaction with HIV infection. Only three studies evaluated the effect of combination antiretroviral therapy (cART) on functional data suggesting an increase in activation with the use of cART. fMRI is a sensitive instrument to detect subtle cognitive changes in HIV patients. Open questions remain regarding the effects of cART on fMRI and the effects of aging on fMRI.

**Electronic supplementary material:**

The online version of this article (doi:10.1007/s13365-016-0483-y) contains supplementary material, which is available to authorized users.

## Introduction

In the recent era of combination antiretroviral therapy (cART), infection with the human immunodeficiency virus (HIV) has changed from a rapidly fatal disease into a chronic condition with subsequent comorbidities (Kirk and Goetz 2009; Murray et al. 2014). One of the most important comorbidities in HIV-infected patients is cognitive decline, resulting in HIV-associated neurocognitive disorders (HAND). It is estimated that around 50 % of all HIV-infected patients has a form of HAND (Heaton et al. 2010). Moreover, in this aging population, cognitive disorders are the most worrying aspect of the disease for the patients themselves. The advances in cART, over the past decades, have led to a shift in prevalence from the most severe form of HAND, HIV-associated dementia (HAD), towards milder forms of neurocognitive disorders like asymptomatic neurocognitive impairment (ANI) and mild neurocognitive disorder (MND) (Heaton et al. 2010; Tan and McArthur 2011; Antinori et al. 2007; McArthur et al. 2010). The large proportion of HIV-infected patients suffering from ANI poses particular challenges for diagnosis, because by definition, these patients do not experience or report symptoms. Diagnosing ANI and other forms of HAND is important, as a recent study showed that patients with ANI have a two- to sixfold increased risk of developing symptomatic cognitive problems as opposed to neurocognitive normal patients (Grant et al. 2014). However, there are some debates on the diagnosis of ANI and whether the neurocognitive decline is not due to other comorbidities (Nightingale et al. 2014). Sensitive screening instruments would therefore be a welcome addition to the diagnostic armamentarium.

Neuropsychological (NP) testing is the primary method for diagnosing HAND. However, this is time consuming and may not be sensitive enough to detect subtle neurocognitive changes, which may underlie the milder forms of HAND such as ANI (Ances and Hammoud 2014). Several studies have shown that blood oxygenated level dependent (BOLD) functional magnetic resonance imaging (fMRI) is more sensitive in detecting abnormal brain function compared to NP testing (Haley et al. 2011; Saykin et al. 1999; Sumowski et al. 2012; Sweet et al. 2006). From 2001 onwards, there have been several studies evaluating the role of fMRI in the detection of neuronal dysfunction in HIV-infected patients, first focusing on attention and motor functions while later studies investigated executive functions and fronto-striatal networks (Ernst et al. 2002; Chang et al. 2001; Schweinsburg et al. 2012; Plessis et al. 2015). In order to determine whether fMRI can be used as a diagnostic tool aiding in HAND diagnosis, it is important to summarize these studies and evaluate their usefulness in terms of applicability, risk of bias, and scientific limitations. A meta-analysis and concise systematic review was published in 2014, mostly focusing on the fronto-striatal system and including different forms of fMRI than BOLD fMRI, the most frequently used form of fMRI (Plessis et al. 2014). To date, however, no extensive systematic review on solely BOLD fMRI, investigating all brain networks, and using only studies with a HIV-negative control group has been published. This can be explained by the fact that BOLD fMRI is a relatively new research tool and, as mentioned before, HIV infection only recently became a chronic infection. In order to properly appraise the utility of fMRI in chronic HIV infection, it is important to extensively outline the available data on this subject. This can serve as a solid fundament for future research on this promising novelty in the field. Therefore, the objective of this review is to systematically analyze studies investigating BOLD fMRI in HIV-positive and -negative subjects in terms of differences in activation patterns, in order to evaluate the effect of HIV infection on brain function and the impact of age and medication.

## Methods

### Search and selection

This systematic review was conducted according to the Preferred Reporting Items for Systemic review and Meta-Analysis (PRISMA) framework. The protocol for this study is included in the international prospective register of systematic reviews PROSPERO under registration number CRD42015015698. Eligibility criteria for the included studies were as follows: (1) conducting with HIV-positive patients, (2) using BOLD fMRI, and (3) including a HIV-negative control group.

A literature search was performed in June 2015 using three online databases: Embase, PubMed, and the Cochrane databases. The search terms are presented in supplementary document 1. Mesh terms were used if available. All time frames were included because of the novelty of fMRI. We included only original research papers in English or in Dutch.

### Study selection

**Fig. 1:**
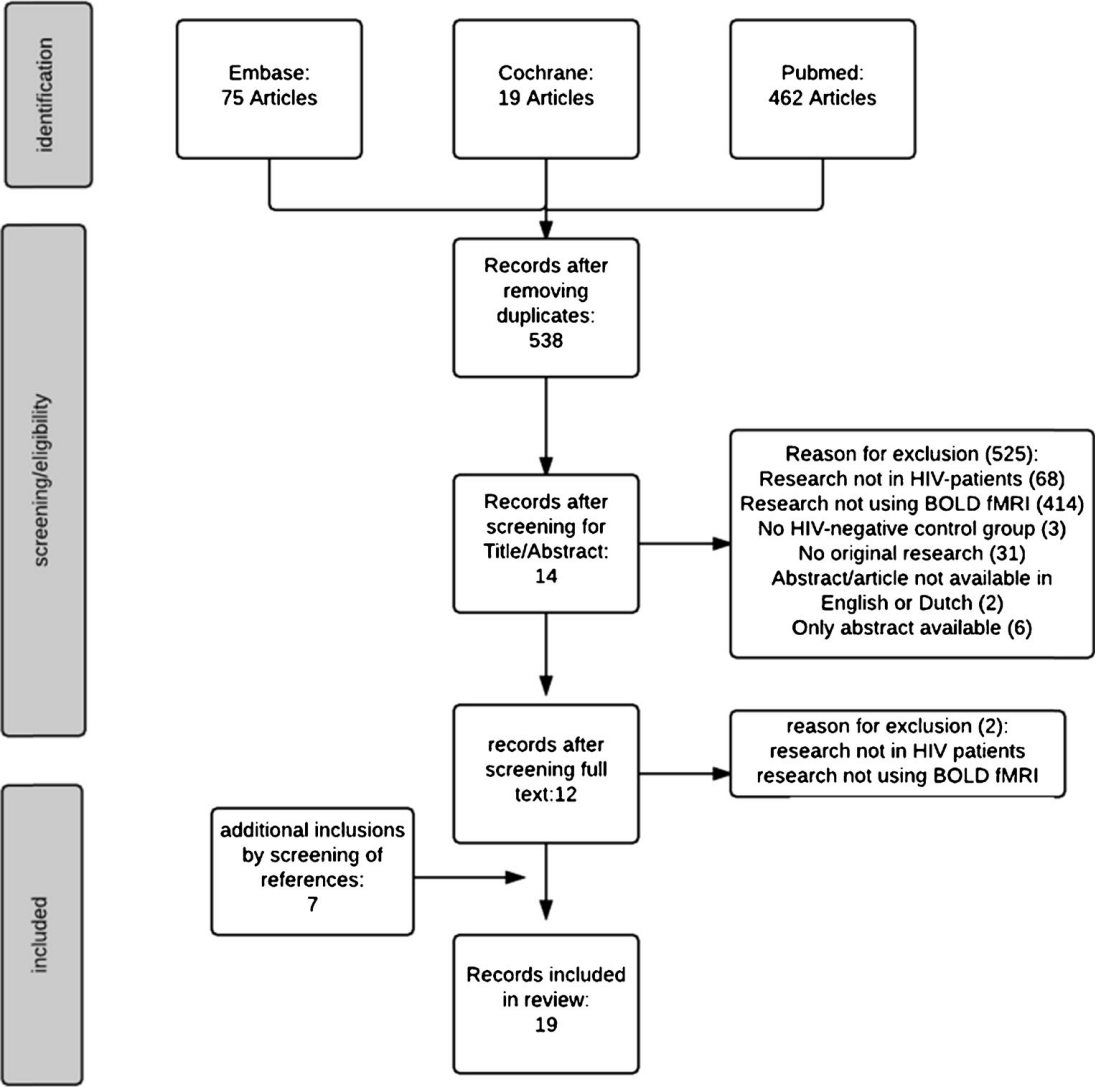
Process of study selection. *BOLD* blood oxygenated level dependent, *fMRI* functional MRI

The first screening of papers for eligibility was done by one author (CH). Duplicates were identified and removed. A total of 538 papers were identified. Full text evaluation of the remaining studies for eligibility was performed independently by two authors (CH and JEA). In addition, references of the identified studies were cross-checked for any additional relevant studies. The process for selecting studies is summarized in Fig. 1. One reference from cross-checking studies was excluded because it was a conference report not published in a core medical journal (Qiu et al. 2011).

### Data extraction and validity

Data extraction was performed by two independent authors (CH and JEA) using a standardized data extraction form. Inconsistencies between study forms were discussed and, when appropriate, reviewed by a third author (MV) for majority decision. Where doubts remained, authors of the original paper in question were contacted. Variables included in the form were study setting, number of patients, patient characteristics including HIV-specific variables, cART use, co-medication, substance abuse, and cognitive status, fMRI task used, form of analysis of fMRI data, and behavioral and fMRI results. Results were expressed as statistically significant differences in activation measured by BOLD signal between HIV-positive and -negative individuals. The statistical inferences used on fMRI data were summarized or simplified; if a multiple comparison correction was used, either by family-wise error or false discovery rate, this was reported, together with the level of correction (voxel or cluster level) and the *p* value used. A risk of bias assessment was performed for each individual study using a standardized risk of bias assessment form (QUADAS-2). In this assessment, we focused on the risk of bias in inclusion and possible confounders and not specifically on risks involved in the statistical inference of fMRI data since the latter information is presented in the result tables.

### Analysis

Medians and standard deviations for baseline characteristics were calculated when needed and when data was available. The results were grouped per form of analysis (whole brain or regions of interest) and furthermore by pairing studies that investigated the effect of HIV infection on the characteristics of the BOLD signal and those who specifically studied the interaction of HIV and aging.

### Role of the funding source

There was no role of the funding source in study design, in collection, analysis, and interpretation of data, in the writing of the report, or in the decision to submit the paper for publication.

## Results

A total of 538 studies were identified after searching Embase, PubMed, and Cochrane databases, of which 12 were eligible for inclusion after screening the title and abstract. Reasons for exclusion and further process of study selection are depicted in Fig. 1. Finally, after cross-checking references of the included studies, another seven publications were included leading to 19 manuscripts in the final selection.

### Study characteristics

A summary of study characteristics is given in Table 1. All studies took place in the USA (Ernst et al. 2002; Chang et al. 2001; Schweinsburg et al. 2012; Caldwell et al. 2014; Thomas et al. 2013; Ances et al. 2011; Ernst et al. 2009; Melrose et al. 2008; Chang et al. 2013; Ances et al. 2008; Chang et al. 2008; Juengst et al. 2007; Maki et al. 2009; Chang et al. 2004; Castelo et al. 2006; Ances et al. 2010a; Ortega et al. 2015; Ipser et al. 2015), except for one, which was situated in South Africa (Plessis et al. 2015). With only two (12 %) longitudinal studies (Ances et al. 2011; Ernst et al. 2009), the majority (78 %) was cross-sectional in design. In total, 19 studies included a total of 573 HIV-positive and 408 HIV-negative patients. Most of the patients were male, with six studies having solely male participants (Ernst et al. 2002; Chang et al. 2001; Melrose et al. 2008; Chang et al. 2013; Chang et al. 2008; Castelo et al. 2006). The mean age of all participants was 41.4 years (95 % CI 41.06–41.64). The majority of HIV patients were on cART with only the South African study having no patients on cART (Plessis et al. 2015). Two studies did not report cART use, and average cART use was 56 % (95 % CI 53–58) in the other studies. Four studies specified the type of cART used and/or gave information on its CNS penetration effectiveness score (Ernst et al. 2002; Chang et al. 2001; Ances et al. 2008; Chang et al. 2008). Seven studies included patients with cognitive deficits ranging from mild impairment to HAD, either according to the former criteria or the new Frascati criteria (Chang et al. 2001; Thomas et al. 2013; Melrose et al. 2008; Chang et al. 2013; Ances et al. 2008; Juengst et al. 2007; Chang et al. 2004).

**Table 1:** Baseline characteristics

Author	Correlated NPA		No.	Mean age (SD)	% male	Years of education (SD)	Impaired cognition	substance abuse	Co-medication	% on cART	Type of cART	Duration of cART	Mean current CD4 (IQR)	Mean nadir CD4 (IQR)	Duration infection	No sign. difference on
Caldwell (2014)	No	HIV+	34	46.1 (8.5)	54	12.6 (1.8)	NR	0	NR	79	NR	NR	550	201	7.6 years	Age sex
		HIV−	28	44.9 (12.7)	65	14.0 (3.4)	NR	0	NR							
Thomas (2013)	Yes	HIV+	52	41 (14)	90	14 (2)	23 % impairment	25 %	NR	44	NR	NR	377 (291–616)	260 (116–386)	NR	Age
		HIV−	52	44 (14)	51	15 (3)	NR	NA	NR							
Ances (2010a)	Yes	HIV+	6	30 (7)	100	15 (2)	GDS 0.34	16 %	NR	83	NR	NR	757 (424–900)	588 (438–750)	NR	Age sex education
		HIV−	10	30 (6)	60	18 (3)	NR	0	NR							
Ernst (2009)	Yes	HIV+	31	49.6 (8.4)	97	15.5 (2.2)	NR	0	No neuro-impairing medication	100	NR	NR	415 (40.4)	152 (24)	NR	Age sex education hematocrit
		HIV−	32	46.9 (13)	88	15.5 (2.3)	NR	0	No neuro-impairing medication							
Melrose (2008)	Yes	HIV+	11	40.8 (7.1)	100	16.3 (1.5)	9 % mild impairment	9 % alcohol	36.4 % anti-depressant	91	NR	9.9 (5.4) years	694.2 (197)	NR	9.9 (5.4) years	Age sex education
		HIV−	11	40.9 (8.7)	100	16.9 (1.8)	0	9 % alcohol	0							
Chang (2013)	Yes	HIV+	66	47.1 (8.6)	100	14.6 (2.3)	43.90 % HAND	0	NR	NR	NR	NR	401.7	158.4	144.22 months	Age sex education hematocrit
		HIV−	56	45.7 (12.7)	100	14.8 (2.2)	0	0	NR							
Ances (2008)	Yes	HIV+	24	45.5 (6.9)	71	14.5 (3.5)	GDS 0.95 of 7 MND en 8 HAD	0	NR	100	1*	20 months	368	NR	>1 year	Age sex education
		HIV−	10	46 (12.6)	60	14 (3.2)	GDS 0.3	0	NR							
Chang (2008)	Yes	HIV+	24	40.15 (8.1)	100	13.7 (2.6)	GCD art+:6.2(0.2) art−:6.0(0.9)	0	No neuro-impairing medication	50	2*	134.8 (21.7) months	467.5	218.5	129.9 months	Age sex education hematocrit
		HIV−	18	39.82 (12.3)	100	13.9 (2.1)	GCD 2.7(0.5)	0	No neuro-impairing medication							
Juengst (2007)	Yes	HIV+	31	47.7 (15.7)	90	14.5 (2.7)	35 % MNCD or HAD	NR	NR	NR	NR	NR	397.41	NR	NR	None
		HIV−	16	42.3 (11.8)	75	14.0 (3.0)	NR	NR	NR							
Ernst (2002)	Yes	HIV+	10	36.3 (7.9)	100	14.8 (2.0)	0	20 % smoking	NR	90	3*	NR	375 (187)	241 (145)	NR	Age sex education
		HIV-	10	36.1 (6.8)	100	15.6 (2.6)	0	20 % smoking	NR							
Chang (2001)	No	HIV+	11	41 (4.8)	100	14 (2.1)	36 % MCMD 27 % mild HIV-dementia	0	NR	91	4*	NR	329 (197)	170 (126)	NR	Age sex education
		HIV−	11	38 (4.8)	100	16.4 (3.3)	0	0	NR							
Maki et al. (2009)	Yes	HIV+	7	41.1	0	11.7	NR	29	NR	43	NR	NR	NR	NR	NR	Age sex education
		HIV−	4	42.8	0	12.3	NR	50	NR							
Chang (2004)	Yes	HIV+	18	38.2 (7)	78	14.2 (1.6)	55 % MCMD 33 % mild dementia	0	No chronic co-medication	83.3	PI-regime	27.7 weeks	287 (36)	123 (37)	91 (15) months	Age sex education hematocrit
		HIV−	18	38.0 (8.8)	78	14.5 (1.9)	0	0	No chronic co-medication							
Castelo (2006)	Yes	HIV+	14	39 (9.1)	100	14.8 (2.0)	0	0	5*	71	NR	NR	690 (370) data from 10 out of 14 patients	NR	NR	Age sex education
		HIV−	14	40 (10.4)	100	16 (1.9)	0	0	5*							
Ances (2010b)	No	HIV+	26	39	77	16	NR	0	NR	60	NR	At least 3 months	486	278	NR	Age sex education
		HIV−	25	41	56	15	NR	0	NR							
Schweinsburg (2012)	No	HIV+	11	41.8 (6.1)	82	13.9 (2.4)	NR	0	NR	91	NR	NR	NR	140 (29–300)	NR	Age sex education
		HIV−	13	42.5 (14.5)	77	14.9 (2.1)	NR	0	NR							
du Plessis (2015)	Yes	HIV+	18	32 (4.6)	11	11 (10–12)^6*^	GDS 0.21	0	NR	0	NA	NA	433 (199)	NR	NR	Age sex education
		HIV−	16	28 (5.2)	6	12 (11–12)^6*^	GDS 0.17	0	NR							
Ipser et al. (2015)	Yes	HIV+	15	40.6 (14.5)	80	13.8 (2.2)	GDS 0.32 (0.28)	Alcohol 13.3 %	NR	86	NR	NR	548 (255.29)	310 (193)	80.4 months	Age sex education
		HIV−	15	39.7 (12.6)	87	13.7 (1.3)	GDS0.29 (0.26)	Alcohol 46.7 %	NR							
Ortega et al. (2015)	Yes	HIV+	131	39.0	72	13.1	*Z*-score −0.39	NR	NR	63	NR	NR	551	281	NR	Sex education
		HIV−	45	31.7 (10.9)	58	13.4 (2.7)	*Z*-score 0.14	NR	NR							

*cART* combination antiretroviral therapy, *CD4* CD4-cell count, *GCD* global cognitive deficit, *GDS* global deficit score, *HAD* HIV-associated dementia, *HIV+* HIV-positive, *HIV−* HIV-negative, *IQR* interquartile range, *MCMD* minor cognitive motor disorder, *NA* not applicable, *NPA* neuropsychological assessment, *NR* not reported, *SD* standard deviation

*(low CPE, high CPE) protease inhibitor 50 %, 42 %; nucleoside reverse transcriptase inhibitor 66 %, 83 %; non-nucleoside reverse transcriptase inhibitor 33 %, 42 %

°8 tenofovir, 5 lamivudine, 3 zidovudine, 2 abacavir, 1 didanosine, 1 stavudine, 2 emtricitabine, 4 efavirenz, 2 nevirapine, 3 lopinavir/ritonavir, 2 ritonavir, 1 saquinavir, 1 atazanavir, 1 fosamprenavir, 1 nevirapine/lopinavir/ritonavir

∆3× d4t/lam/kaletra, 1× d4t/nelfinavir/nevirapine, 2× d4t/lam/nelfinavir, 1× d4t/rit/saquinavir, 1× azt/lam/capavirine, 1× azt/lam/indinavir

¤1× D4T/ddI, 1× D4T/lamivudine, 2× AZT/lamivudine/indinavir, 1× AZT/3TC/d4T, 1× D4T/nelfinavir/nevirapine, 1× D4T/ininavir/3TC, 1x ritonavir/saquinavir/D4T/3TC 3× ddI/ritonavir/indinavir/D4T/saquinavir

¥2 patients used medication that might affect cognition (effexor, celexa, ambien)

### Critical appraisal

All studies were appraised for risk of bias on four items (patient selection, index test, reference standard, and flow and timing) and for applicability on three items (patient selection, index test, and reference standard) (supplementary document 2). There were four studies that did not use a reference standard (NPA) and therefore could not be completely assessed (Chang et al. 2001; Schweinsburg et al. 2012; Juengst et al. 2007; Castelo et al. 2006; Ances et al. 2010a). The lack of a reference standard would normally be an issue; however, it seems surmountable in this setting where the index test, i.e., fMRI, might be more sensitive than the reference standard. There were only four studies (24 %) that scored inadequate on more than one item, indicating that the risk of bias on the aforementioned items seems low across studies (Schweinsburg et al. 2012; Caldwell et al. 2014; Chang et al. 2013; Chang et al. 2008).

### Impact of HIV on BOLD characteristics

First, studies investigating the influence of HIV infection on the shape or the response of the BOLD signal were analyzed, since HIV replication in the brain has been shown to cause alterations in brain metabolism which might influence signal intensity (Roc et al. 2007). The BOLD signal depends on the hemodynamic response of the brain, which causes a greater delivery of oxygen-rich blood to active neurons as opposed to inactive neurons. Four studies investigated the effect of HIV on the characteristics of the BOLD signal (Ances et al. 2011; Juengst et al. 2007; Ances et al. 2010a; Ances et al. 2010b). Two of them found no significant difference in mean peak values of the hemodynamic response function (an indication of the shape and amplitude of the BOLD signal) when using a motor task in HIV-positive and HIV-negative subjects (Table 2) (Ances et al. 2008; Juengst et al. 2007). The third study, by Ances et al. (2010a, b), using a visual task, found reduced functional changes in BOLD signal in the visual cortex in HIV-positive subjects as opposed to HIV-negative subjects (Ances et al. 2010a). Finally, using the same task, Ances et al. (2011) found in a subsequent study a statistically significant decrease in BOLD signal after 1 year in the HIV-positive group (Ances et al. 2011). It must be noted, however, that the groups used for this latter analysis were rather small (six HIV-positive versus ten HIV-negative subjects). When studying fluctuations in greater detail, it is important to consider the effect of different activation in different subgroups (Rosenblatt et al. 2014) that would require a larger study population. Taken together, the data from these four studies suggest no clear impact of HIV on the characteristics of the BOLD signal, indicating that differences in BOLD response between HIV-positive and -negative participants can be interpreted as a difference in brain activation, i.e., in the amount of neurons activated in a certain region.

**Table 2:** BOLD characteristics

Study	HIV/SN	Region	Task	Software	Threshold	Correction M.C.	Results
Ances et al. (2011)	6/10	Visual cortex	Checker board	NR	*p* = 0·05	Mask used	HIV + showed reduction in mean functional BOLD changes over time and greater inter-subject variance in BOLD measures
Ances et al. (2008)	24/10	Motor	Checker board + squeezing	Voxbo	NA (amplitude BOLD signal)	NA	No significant difference in BOLD amplitude between HIV + and −
Juengst et al. (2007)	31/16	HRF	Finger tapping	NR	NA (HRF)	NA	No significant difference in mean peak values between HIV + and HIV−
Ances et al. (2010a)	26/25	Visual cortex	Checkerboard	AFNI	*p* = 0·05	Yes, not specified	HIV+ reduced functional changes in BOLD signal

*AFNI* analysis of functional neuroimages, *BOLD* blood oxygenated level dependent, *HIV* HIV-positive patients, *HRF* hemodynamic response function, *M.C.* multiple comparisons, *NA* not applicable, *NR* not reported, *SN* seronegative controls

### Whole-brain and regions of interest analyses

We analyzed 15 studies focusing on either whole-brain or region of Interest (ROI) analyses in HIV-positive and -negative subjects (Tables 3 and 4) (Ernst et al. 2002; Chang et al. 2001; Schweinsburg et al. 2012; Plessis et al. 2015; Caldwell et al. 2014; Thomas et al. 2013; Ernst et al. 2009; Melrose et al. 2008; Chang et al. 2013; Chang et al. 2008; Maki et al. 2009; Chang et al. 2004; Castelo et al. 2006; Ortega et al. 2015; Ipser et al. 2015). While whole-brain analyses are used to explore effects throughout the brain, ROI analyses focus on predefined regions, either anatomically or by a previous independent study, thereby reducing type I error. However, when regions are not previously specified but rather defined based on whole-brain results from the same study, the chance of bias is drastically increased (Kriegeskorte et al. 2009). Neuropsychological studies have suggested that brain regions involved in attention, working memory, and episodic memory may be particularly affected in HIV-positive patients with HAND (Heaton et al. 2011; Reger et al. 2002; Grant 2008). More recent neuroimaging studies center on fronto-striatal circuits.

**Table 3:** Whole-brain analysis of difference BOLD signal HIV−/+ patients

Study	HIV/SN	Network	Task	Software	Statistical thresholding	Result
Caldwell et al. (2014)	34/28	Working memory	Sequential letter task	FEAT	FWE corrected at voxel level *p* < 0.05 later relaxed (not specified)	HIV+ greater activation on the simpler attention task but less activation on the working memory task
Ernst et al. (2009)	31/32	Attention	Tracking balls	SPM2	FWE corrected at voxel level *p* < 0.05	HIV+ more activation in right prefrontal region only with the most difficult task
Melrose et al. (2008)	11/11	Semantic event sequencing	Picture sequencing task + object discrimination control	SPM2	Voxel threshold 0.001 uncorrected, small volume correction	HIV+ less signal change in frontal regions and left caudate and more signal changes in postcentral/supramarginal gyrus
						Functional connectivity: dysfunction within the basal ganglia and prefrontal cortex and within interactions between these regions
Chang et al. (2013)	66/56	Attention	Tracking balls	SPM8	FWE corrected at cluster level *p* < 0.05	HIV+ has load-dependent decreased activation in right temporal region, while HIV− showed load-dependent increase
Chang et al. (2008)	24/10	Attention	Tracking balls	SPM2	FWE corrected at cluster level *p* < 0.05 used various thresholds	HIV+ has greater load-dependent activation in right frontal and cingulate regions
Ernst et al. (2002)	10/10	Working memory	Sequential letter task	SPM99b	Voxel threshold 0.001 uncorrected	HIV+ has more BOLD activation in the lateral prefrontal cortex on all tasks
Chang et al. (2001)	11/11	Working memory	Sequential letter + number task	SPM99b	Voxel threshold 0.001 uncorrected	HIV+ has greater activation in parietal regions and frontal lobes (lateral prefrontal cortex and supplementary motor area)
Maki et al. (2009)	7/4	Memory	Encoding task, recognition task	SPM2	Cluster corrected (min size >30) uncorrected threshold *p* < 0.05	Encoding: HIV− more activation in hippocampal and temporal/frontal cortical structures. Recognition: HIV+ more in left superior temporal gyrus, hippocampus, and right insular cortex
Chang et al. (2004)	18/18	Attention	Tracking balls	SPM99b	Cluster corrected for M.C. (not specified)	HIV+ decreased activation in the normal visual attention network and increased activation in adjacent/contralateral structures
Castelo et al. (2006)	14/14	Memory	Encoding + recognition task	SPM99b	NR	Encoding: no difference. Recognition: HIV+ less activity in right posterior hippocampus, right inferior frontal gyrus, and left lingual gyrus and more activity in lateral frontal and posterior parietal regions
Schweinsburg et al. (2012)	11/13	Fronto-striatal	Mental rotation task	AFNI	Cluster corrected multiple thresholds/cluster size	HIV+ had increased activation in areas of the PPC-striato-frontal pathway and in left insular and right occipital cortex and less activation in the anterior cingulate
Plessis et al. (2015)	18/16	Ventral-striatal	Reward task	SPM8	FWE corrected at cluster level *p* = 0.05	No between group differences

*AFNI* analysis of functional neuroimages, *BOLD* blood oxygen level dependent, *FEAT* fMRI expert analysis tool, *FWE* family-wise error, *M.C.* multiple comparisons, *NR* not reported, *SPM* statistical parametric mapping, *PPC* postero-parietal cortex

**Table 4:** ROI analysis of differences BOLD signal HIV+/− patients

Study	HIV/SN	Network	Task	Software	Corrected for MC?	Pre-specified ROI?	Results
Thomas et al. (2013)	52/52	Functional connectivity 5 domains	Resting state	NR	FDR corrected *p* < 0.05	Yes	HIV+ had less intra- and internetwork correlations in several functional brain networks
Chang et al. (2008)	24/18	Visual attention	Tracking balls	SPM2	Uncorrected *p* = 0.05	No	HIV+ load-dependent increase in frontal regions when HIV− has load-dependent decrease
Maki et al. (2009)	7/4	Episodic encoding	Encoding task, recognition task	SPM2	Cluster corrected	Yes	HIV+ decreased hippocampal activity during encoding and increased hippocampal activation during recognition
Castelo et al. (2006)	14/14	Episodic encoding	Encoding task, recognition task	SPM99b	Not reported	Both	HIV+ had attenuated activation of brain regions known to support episodic encoding (right posterior hippocampus, left and right lingual gyrus, right inferior frontal gyrus) and recruited additional cortical regions
					Hippocampal activation; no	Yes	HIV+ less activation in bilateral hippocampus
Plessis et al. (2015)	18/16	Ventral-striatal reward	Reward task	SPM8	no	Yes	HIV+ decrease in activation in ventral striatum for anticipating neutral and rewarding cues
Ortega et al. (2015)	132/49	Functional connectivity 4 domains	Resting state	FS-FAST	FDR corrected *p* = <0.05	Yes	HIV+ had lower cortico-striatal functional connectivity. HIV+ cART+ had higher connectivity than HIV+ cART−
Ipser et al. (2015)	15/15	Functional connectivity 3 domains	Resting state	AFNI	Not reported	Yes	HIV+ had reductions in connectivity in fronto-striatal regions.

*AFNI* analysis of functional neuroimages, *FDR* false discovery rate, *FS-FAST* freesurfer functional analysis stream, *MC* multiple comparisons, *NR* not reported, *ROI* region of interest, *SPM* statistical parametric mapping

Four of the 15 fMRI studies focused on attention deficits using visual attention tasks (Ernst et al. 2009; Chang et al. 2013; Chang et al. 2008; Chang et al. 2004). In these tasks, subjects had to track a certain number of balls among the other moving balls. Overall, studies reported an increase in activation in the attention network (right (pre)frontal and cingulate regions) and/or adjacent structures when attentional load increased. HIV-positive patients performed at the same behavioral level (test accuracy and reaction time) as HIV-negative subjects up until the most difficult tasks. Taken together, these data suggest that HIV-positive patients show hyperactivation of brain regions and/or recruit adjacent regions to achieve the same behavioral results, up onto the point where functional brain activation falls short and behavioral results are affected. Apparently, more neural activation is needed in the HIV-positive individuals. These analyses suggest that in HIV-positive subjects, an attention deficit is present which can, to a certain degree, be counter balanced by the use of brain reserve capacity (Bosch et al. 2010).

Three studies employed working memory paradigms. Working memory was tested using a sequential number task, in which a series of numbers is presented and subjects were instructed to press a button when the number shown is the same as *n* items before. Ernst et al. (2002) and Chang et al. (2001) found an increase in activation in the lateral prefrontal cortex and/or parietal regions in the HIV-positive group (Ernst et al. 2002; Chang et al. 2001). Caldwell et al. (2014) found that HIV-infected subjects had more activation but similar accuracy on the simpler tasks but less activation and diminished accuracy on the more difficult tasks, when compared to HIV-negative controls (Caldwell et al. 2014).

In addition to the studies investigating attention and working memory, there were two studies investigating memory (encoding and recall) (Maki et al. 2009; Castelo et al. 2006). Maki et al. (2009) and Castelo et al. (2006) used comparable tasks, in which subjects were instructed to remember either words or pictures and recall them later. Whole-brain as well as ROI analyses revealed differences for HIV-positive patients in activation in hippocampal and/or temporal/frontal cortical structures. Castelo et al. (2006) found no difference in activity during encoding and less activity during recognition for HIV-positive patients, while Maki et al. (2009) found less activity during encoding and more activity during recognition in HIV-positive patients. These conflicting results could possibly be due to the small sample size of both studies (*n* = 11^29^ and *n* = 28^31^) and/or the fact that the task used differed slightly. Furthermore, Castelo et al. (2006) did not provide insight in the statistical inference used, which makes it more difficult to interpret their outcomes. In all, despite the limitations, all memory studies do suggest a dysfunction of hippocampal-prefrontal regions in HIV-positive subjects, possibly underlying memory deficits.

Four studies centered on the fronto-striatal network. This is important as frequently occurring symptoms in HAND like changes in executive functioning suggest a dysfunction in this circuit (Reger et al. 2002; Sahakian et al. 1995; Wiley et al. 1998). Moreover, a recent meta-analysis found evidence for hyperactivation in the fronto-striatal circuit in HIV-positive subjects (Plessis et al. 2014). Melrose et al. (2008) used a semantic event sequencing task, during which subjects had to arrange semantic events in the right order (Melrose et al. 2008). They found more activation in the right postcentral/supramarginal gyrus for the HIV-positive group, while the HIV-negative groups showed more activation in the frontal regions. Functional connectivity analyses on resting state data by Melrose et al. (2008), Thomas et al. (2013), Ortega et al. (2015), and Ipser et al. (2015) suggested dysfunction between basal ganglia and other (frontal) regions and less intra- and internetwork correlations in certain prespecified brain networks (Thomas et al. 2013; Melrose et al. 2008; Ortega et al. 2015; Ipser et al. 2015). This means that even without using a task, a disturbance could be found between networks in HIV-positive subjects compared to seronegatives. Schweinsburg et al. (2012) studied the effect of HIV on mental rotation, because it is part of the fronto-striatal circuit (Olesen et al. 2007). They found increased activation in areas of the postero-parietal cortex pathway and in the left insular and right occipital cortex, together with less activation in the anterior cingulate in HIV-positive subjects (Schweinsburg et al. 2012). Reaction times and accuracy on the fMRI tasks did not differ between the two groups. Finally, a study by du Plessis et al. (2014) on fronto-striatal reward processing included only cART naïve subjects. Using a whole-brain analysis, the study found no significant difference between cART naïve HIV-negative and -positive subjects. However, a ROI analysis revealed significant less activation in the ventral striatum during anticipating neutral or rewarding cues in the latter group. They did not report differences in the frontal function.

### Effect of cART

Only two studies compared functional data between patients with and without cART (Chang et al. 2008; Ortega et al. 2015) and one who investigated differences in BOLD signal for different kinds of cART (Ances et al. 2008). The two papers studying attention both found a significant difference in BOLD activation with a greater attentional load-dependent increase in brain activation for patients on cART and lower accuracy on the performance of the most difficult task (Ances et al. 2008; Chang et al. 2008). Ances et al. (2008) found an increase in the BOLD response for patients on low CNS penetration effectiveness drugs (Ances et al. 2008). Ortega et al. (2015) found higher functional connections in HIV patients with cART then HIV patients without cART in fronto-striatal networks using a functional connectivity analysis.

### Effect of aging

Finally, six studies report on the effect of aging on brain function in HIV-positive and HIV-negative subjects (Table 5) (Thomas et al. 2013; Ernst et al. 2009; Chang et al. 2013; Juengst et al. 2007; Ances et al. 2010a; Ipser et al. 2015). Since HIV patients are aging, it is important to investigate if aging has an interaction with HIV status on functional data because both HIV and aging have a degenerative effect on the brain and functional brain regions. Two studies investigated the effect of HIV and aging on characteristics of the BOLD signal and found no interactions (Juengst et al. 2007; Ances et al. 2010a). Thomas et al. (2013) and Ipser et al. (2015) calculated functional connectivity during a resting state to evaluate regional interactions between prespecified functional networks. They found similar decreases in correlations between networks with aging and HIV infection, but there was no interaction between HIV and aging (Thomas et al. 2013; Ipser et al. 2015). Ernst et al. (2009) and Chang et al. (2013) used the same visual attention task (Ernst et al. 2009; Chang et al. 2013). The longitudinal study by Ernst et al. (2009) found that after 1 year follow-up, HIV-positive subjects had more activation in the right prefrontal and posterior parietal cortices and bilateral cerebellum than HIV-negative subjects (Ernst et al. 2009). A possible explanation is a learning effect in the HIV-negative group or an effect of ongoing brain injury in the HIV-positive group. Chang et al. (2013) also reported interactions between age and HIV status with greater age-related increase in activation in various regions (Chang et al. 2013). Noting that from the five studies, only one found an interaction with HIV and aging; the limited evidence seems to point towards there being no interaction between these parameters. We therefore decided to regard them as independent factors.

**Table 5:** BOLD signal differences HIV+/− patients combined with aging effect

Study	HIV/SN	Network	Task	Software	WB/ROI	Statistical inference	Results
Thomas et al. (2013)	52/52	Functional connectivity 5 domains	Resting state functional connectivity	NR	ROI	FDR corrected threshold of 0.05	Aging causes decrease in intranetwork correlations in DMN and SAL and internetwork correlations between DMN-SAL. No interaction between HIV and aging
Ernst et al. (2009)	31/32	Visual attention	Tracking balls	SPM2	ROI	FWE corrected at voxel level *p* < 0.05	After 1 year, HIV+ more BOLD signal in right prefrontal and posterior parietal cortices and cerebellum bilaterally. HIV− less BOLD signal after 1 year
Chang et al. (2013)	66/56	Visual attention	Tracking balls	SPM8	WB	FWE corrected at cluster *p* < 0.05	HIV+ had greater age-related increases in brain activation in right parietal, cingulate and paracentral regions, cerebellar vermis, left frontal, temporal and occipital regions
Juengst et al. (2007)	31/16	HRF	Finger tapping	NR	WB	NA (HRF)	No effect or interaction with HIV status for age in mean BOLD peak value
Ances et al. (2010b)	26/25	Visual cortex	Checkerboard	AFNI	VOI	*p* = 0.05 corrected for M.C. (not specified)	HIV and increasing age independently caused decreases in functional BOLD signal, no interaction
Ipser et al. (2015)	15/15	Functional connectivity 3 domains	Resting state	AFNI	ROI	Not reported	Reduction in connectivity in individuals over 50 years, no interaction between age and HIV

*AFNI* analysis of functional neuroimages, *BOLD* blood oxygen level dependent, *DMN* default mode network, *FDR* false discovery rate, *FWE* family-wise error, *HRF* hemodynamic response function, *M.C.* multiple comparisons, *NR* not reported, *ROI* region of interest analysis, *SAL* salience network, *SPM* statistical parametric mapping, *VOI* volume of interest analysis, *WB* whole-brain analysis

## Discussion

This systematic review of 17 studies describes the effect of HIV infection on brain function as measured by BOLD fMRI. Overall, HIV does not seem to alter BOLD characteristics. This is important, as this finding suggests that the coupling between neural activation and the BOLD response itself is not necessarily different in HIV-positive patients. A difference in BOLD response is therefore attributable to a difference in the amount of neural activation. The majority of studies found that for completing the same task, HIV-positive patients showed more activation or recruited more regions when compared to HIV-negative controls. Although there is a large variety in the study design, studied populations, and levels of statistic inferences, most evidence seems to point to affected fronto-striatal function. There appears to be no or limited interaction between HIV status and aging on functional neuroimaging data, although there are few longitudinal studies. Finally, the effect of cART on brain function is not yet been adequately addressed.

Since its introduction in the 1990s, fMRI has been proven to be a very sensitive instrument, with an even greater ability to detect functional brain abnormalities than neuropsychological assessment (Haley et al. 2011; Saykin et al. 1999; Sumowski et al. 2012). Neuropsychological studies have shown that HIV seems to predominantly affect the fronto-striatal network (Grant 2008; Ellis et al. 2007; Woods et al. 2009). This network consists of neural pathways that connect frontal regions with the basal ganglia, and these circuits are, among other things, involved in executive functioning (Plessis et al. 2014; Grant 2008; Watkins and Treisman 2015). This systematic review confirms these neuropsychological test observations by showing that impairment of the fronto-striatal system was more pronounced in HIV-positive versus HIV- negative patients. This is consistent with previous literature (Plessis et al. 2014). It is important to note that studies in this review suggest that, even without clinical symptoms or neuropsychological abnormalities, a functional impairment exists in the brains of HIV patients. One explanation for this occurrence is the so-called brain reserve theory, where patients use a hyperactivation or activation of adjacent structures, thus more neural effort to achieve the same behavioral results (Holt et al. 2012). Compared to controls, HIV-positive participants show an overall comparable behavioral performance though performance in behavioral outcomes is poor for the more difficult tasks.

It appears that HIV patients use hyperactivation of brain regions and recruitment of additional brain regions to maintain the same behavioral score, but this mechanism falls short when performing the more difficult tasks. It appears that this hyperactivation is inefficient, possibly due to interfering processes related to the HIV infection. There are several theories of how HIV infection results in functional impairment: first of all, the virus itself, which enters the central nervous system (CNW) within days after infection (Davis et al. 1992; González-Scarano et al. 2005). There is no evidence that HIV actually infects or damages neurons, but due to specific viral proteins produced by infected cells such as gp120, Tat, or Vpr, subsequent local damage can be done (Price et al. 1988). The neurotoxicity theory by HIV is supported by the fact that starting combination antiretroviral therapy often greatly improves the cognitive ability of patients suffering from HAD (Price and Spudich 2008). However, even in patients receiving adequate antiretroviral therapy, cognitive decline can still occur (McArthur et al. 2004). Perhaps, the compartmentalization of HIV in the CNS and the accompanying local ongoing neuro-inflammation or the sensitizing of the immune system by the virus might be an explanation for this observation (Schouten et al. 2011; Campillo-Gimenez et al. 2014). Furthermore, the effect of the virus has been compared to the neurodegenerative process seen in aging. However, the four papers in this systematic review investigating aging and HIV suggest that there is no or limited interaction between HIV status and aging and that they are independent factors to consider.

Antiretroviral drugs are another potential important cause for cognitive disorders in HIV-positive patients. With the recently published INSIGHT START study in mind, it is recommended to start cART even in patients with CD4-counts above 500 cells per cubic millimeter (Initiation of Antiretroviral 2015). Subsequently, this will lead to more patients on therapy and therefore it thus remains of importance to investigate the (sub)clinical and possible long-term consequences of continual antiretroviral therapy on cognition. One of the drugs often implicated in decreased cognitive functioning, is Efavirenz (Ciccarelli et al. 2011). For example, a recent study on treatment interruption found an improvement in cognition as measured by neuropsychological assessment (NPA) after cessation of therapy (Robertson et al. 2010). Additionally, the authors found a difference in improvement after discontinuing different cART regimes, with cessation of Efavirenz containing regimes giving the most effect. Indeed, the effect of cART on cognitive performance has been described before, with Efavirenz as the most significant example (Clifford et al. 2005). Studies in this review showed that patients on cART use more of their brain reserve, and the type of cART affects the BOLD response. This suggests a possible effect of medication and the type of medication on cognition in HIV patients. Based on the results of this systematic review, functional MRI appears to be an appropriate tool to detect subtle cognitive changes. There are, however, very few studies investigating the effects of chronic cART on the CNS. Recently, another South African study in cART naïve HIV-positive patients investigating the fronto-striatal network using an inhibition task was published, showing subcortical dysfunction (du Plessis et al. 2015). Currently, a randomized longitudinal study is underway utilizing fMRI to estimate the effect of Efavirenz on cognition (clinicaltrials.gov NCT02308332).

Another important consideration in this review is the used of statistical and analytical methods used in various studies. First, most studies included only small numbers of patients sometimes hampering firm conclusions. Another problem is statistical inference. Following improvements in fMRI analysis techniques and software, statistical and methodological issues have become less of a problem during recent years. For example, the earliest studies did not properly correct for multiple comparisons (Ernst et al. 2009; Chang et al. 2004; Castelo et al. 2006) or proper thresholding (Chang et al. 2008; Castelo et al. 2006) while more recently published studies tend to have better methodological quality (Plessis et al. 2015; Thomas et al. 2013; Chang et al. 2013). Another limitation is that studies included in this review all described a very “clean” population, i.e., dominantly male participants, with ages between 30 and 50, and lacking information on comorbidities, co-infections, or previous cART regimes. Therefore, no conclusions could be drawn regarding the effect of these factors on functional brain imaging. It is important for future fMRI studies to include younger patients or those with comorbidities or co-medication. Furthermore, different tasks used in these studies make generalizability of results more difficult and need to be addressed in future study designs. The use of longitudinal studies is mandatory since they can aid in exploring the use of fMRi in detecting early changes before clinical symptoms.

Summarizing, when compared with HIV-negative subjects, HIV-positive patients showed a hyperactivation of brain regions, suggesting a so-called brain reserve theory, when investigating regions involved in attention, (working) memory, and executive functioning, with the most evidence pointing to defects in fronto-striatal pathways. Increasing age has a comparable effect on brain function, but it does not interact with HIV status. Limited data points to an effect of cART on brain function. Further research is needed to confirm this effect.

In conclusion, fMRI is a sensitive instrument to detect changes in brain activation associated with subtle cognitive changes in HIV patients.

## Electronic supplementary material

ESM 1 (DOCX 13 kb)

ESM 2 (DOCX 39 kb)
