# Supplementary material for: Review of functional MRI in HIV: effects of aging and medication
Source: J Neurovirol. 2016 Oct 7;23(1):20–32. doi: 10.1007/s13365-016-0483-y (PMC5329077; doi:10.1007/s13365-016-0483-y)
Supplement: Supplementary file 1 — (DOCX 13 kb) [file 13365_2016_483_MOESM1_ESM.docx]

Sup. Document 1: search terms

HIV, HIV patients, human immunodeficiency virus, human immunodeficiency viruses, AIDS virus, acquired immunodeficiency virus, acquired immunodeficiency viruses, HIV-infected patient, HIV infected patient, HIV-infected patients, HIV infected patients, HIV-infected, HIV infected, AIDS, acquired immunodeficiency syndrome, acquired immunodeficiency syndromes, AIDS viruses, HIV infection, HIV-infection, people living with HIV, PLWH, HIV, Acquired Immunodeficiency Syndrome, fMRI, functional MRI, BOLD, blood oxygenated level dependent, functional magnetic resonance imaging, functional magnetic resonance image, functional imaging, blood oxygenated level dependent MRI, BOLD MRI, BOLD fMRI, HAND, HIV-associated neurocognitive disorder, HIV-associated neurocognitive disorders, HIV associated neurocognitive disorder, HIV associated neurocognitive disorders, HIV dementia, HIV-dementia, HIV related cognitive impairment, HIV-related cognitive impairment, AIDS dementia complex, HIV associated dementia, HIV-associated dementia, HAD, HIV encephalopathy, HIV-encephalopathy, ANI, asymptomatic neurocognitive impairment, asymptomatic neurocognitive impairments, MND, mild neurocognitive disorder, mild neurocognitive disorders, AIDS encephalopathy, and AIDS-encephalopathy.
