# Supplementary material for: Review of functional MRI in HIV: effects of aging and medication
Source: J Neurovirol. 2016 Oct 7;23(1):20–32. doi: 10.1007/s13365-016-0483-y (PMC5329077; doi:10.1007/s13365-016-0483-y)
Supplement: Supplementary file 2 — (DOCX 39 kb) [file 13365_2016_483_MOESM2_ESM.docx]

Supplementary document 2: Results risk of bias appraisal of included studies

| study | patient selection –risk of bias | patient selection - applicability | index test – risk of bias | index test - applicability | reference standard – risk of bias | reference standard - applicability | flow and timing | more than 1 unclear/ high | reference standard |
| --- | --- | --- | --- | --- | --- | --- | --- | --- | --- |
| Caldwell 2013^1^ | low | low | high | low | unclear | unclear | unclear | yes | yes |
| Thomas 2013^2^ | unclear | low | low | low | low | low | low | no | yes |
| Ances 2010^3^ | low | low | low | low | low | low | low | no | yes |
| Ernst 2009^4^ | unclear | low | low | low | low | low | low | no | yes |
| Melrose 2008^5^ | low | low | low | low | low | low | low | no | yes |
| Chang 2013^6^ | unclear | low | low | low | low | low | unclear | yes | yes |
| Ances 2008^7^ | low | low | low | low | low | low | low | no | yes |
| Chang 2008^8^ | unclear | low | high | low | low | low | low | yes | yes |
| Juengst 2007^9^ | low | low | low | low | na | na | na | no | no |
| Ernst 2002^10^ | low | low | low | low | low | low | low | no | yes |
| Chang 2001^11^ | unclear | low | low | low | na | na | na | no | no |
| Maki 2009^12^ | unclear | low | low | low | low | low | low | no | yes |
| Chang 2004^13^ | unclear | low | low | low | low | low | low | no | yes |
| Castelo 2006^14^ | low | low | low | low | na | na | na | no | no |
| Ances 2010^15^ | unclear | low | low | low | na | na | na | no | no |
| Schweinsburg 2012^16^ | unclear | unclear | high | low | na | na | na | yes | yes |
| Du Plessis 2015^17^ | low | low | low | low | low | low | low | no | yes |
| Ortega 2015^18^ | low | low | low | low | low | low | low | no | yes |
| Ipser 2015^19^ | low | low | low | low | low | low | unclear | no | yes |

1 Caldwell JZK, Gongvatana a, Navia B a, *et al.* Neural dysregulation during a working memory task in human immunodeficiency virus-seropositive and hepatitis C coinfected individuals. *J Neurovirol* 2014; **20**: 398–411.

2 Thomas JB, Brier MR, Snyder AZ, Ances BM. Pathways to neurodegeneration Effects of HIV and aging on resting-state functional connectivity. *Neurology* 2013; **80**: 1186–93.

3 Ances B, Vaida F, Ellis R, Buxton R. Test-retest stability of calibrated BOLD-fMRI in HIV- and HIV+ subjects. *Neuroimage* 2011; **54**: 2156–62.

4 Ernst T, Yakupov R, Nakama H, *et al.* Declined neural efficiency in cognitively stable human immunodeficiency virus patients. *Ann Neurol* 2009; **65**: 316–25.

5 Melrose RJ, Tinaz S, Castelo JMB, Courtney MG, Stern CE. Compromised fronto-striatal functioning in HIV: an fMRI investigation of semantic event sequencing. *Behav Brain Res* 2008; **188**: 337–47.

6 Chang L, Holt JL, Yakupov R, Jiang CS, Ernst T. Lower cognitive reserve in the aging human immunodeficiency virus-infected brain. *Neurobiol Aging* 2013; **34**: 1240–53.

7 Ances BM, Roc AC, Korczykowski M, Wolf RL, Kolson DL. combination antiretroviral therapy modulates the blood oxygen level-dependent amplitude in human immunodeficiency virus-seropositive patients. 2010; **14**: 1–10.

8 Chang L, Yakupov R, Nakama H, Stokes B, Ernst T. Antiretroviral treatment is associated with increased attentional load-dependent brain activation in HIV patients. *J Neuroimmune Pharmacol* 2008; **3**: 95–104.

9 Juengst SB, Aizenstein HJ, Figurski J, Lopez OL, Becker JT. Alterations in the hemodynamic response function in cognitively impaired HIV/AIDS subjects. *J Neurosci Methods* 2007; **163**: 208–12.

10 Ernst T, Chang L, Jovicich J, Ames N, Arnold S. Abnormal brain activation on functional MRI in cognitively asymptomatic HIV patients. *Neurology* 2002; **59**: 1343–9.

11 Chang L, Speck O, Miller EN, *et al.* Neural correlates of attention and working memory deficits in HIV patients. *Neurology* 2001; **57**: 1001–7.

12 Cohen MH, Weber K, Little DM, *et al.* Impairments in memory and hippocampal function in HIV-positive vs HIV-negative women: a preliminary study. *Neurology* 2009; **72**: 1661–8.

13 Chang L, Tomasi D, Yakupov R, *et al.* Adaptation of the attention network in human immunodeficiency virus brain injury. *Ann Neurol* 2004; **56**: 259–72.

14 Castelo JMB, Sherman SJ, Courtney MG, Melrose RJ, Stern CE. Altered hippocampal-prefrontal activation in HIV patients during episodic memory encoding. *Neurology* 2006; **66**: 1688–95.

15 Ances BM, Vaida F, Yeh MJ, *et al.* HIV infection and aging independently affect brain function as measured by functional magnetic resonance imaging. *J Infect Dis* 2010; **201**: 336–40.

16 Schweinsburg BC, Scott JC, Schweinsburg AD, *et al.* Altered prefronto-striato-parietal network response to mental rotation in HIV. *J Neurovirol* 2012; **18**: 74–9.

17 Plessis S du, Vink M, Joska JA, *et al.* HIV infection results in ventral-striatal reward system hypo-activation during cue processing. *AIDS* 2015; published online June 18. DOI:10.1097/QAD.0000000000000680.

18 Ortega M, Brier MR, Ances BM. Effects of HIV and combination antiretroviral therapy on cortico-striatal functional connectivity. *AIDS* 2015; **29**: 703–12.

19 Ipser JC, Brown GG, Bischoff-Grethe A, *et al.* HIV infection is associated with attenuated frontostriatal intrinsic connectivity: a preliminary study. *J Int Neuropsychol Soc* 2015; **21**: 203–13.
